# Neuropathic Symptoms and Persistent Pain After Hospitalization for COVID-19: An 8-Month Longitudinal Follow-Up Study

**DOI:** 10.3390/healthcare14101300

**Published:** 2026-05-11

**Authors:** Murat Baloğlu

**Affiliations:** Division of Physical Therapy and Rehabilitation, Gazi Yaşargil Training and Research Hospital, Health Science University, Diyarbakır 21280, Turkey; murbal21@hotmail.com

**Keywords:** post-COVID syndrome, neuropathic pain, long COVID, LANSS, visual analog scale

## Abstract

**Background:** Persistent pain, neuropathic symptoms, dyspnea, and impaired quality of life are common components of post-COVID syndrome. However, the long-term trajectory of these symptoms and the factors associated with persistent pain remain incompletely understood. **Methods:** This longitudinal cohort study included 80 patients previously hospitalized with COVID-19. Participants were evaluated at approximately 1, 4, and 8 months after discharge. Pain severity was assessed using the Visual Analog Scale (VAS), neuropathic symptoms using the Leeds Assessment of Neuropathic Symptoms and Signs (LANSS) scale, dyspnea using the modified Medical Research Council (mMRC) scale, functional limitation using the Post-CO9VID Functional Status (PCFS) scale, and health-related quality of life using the EQ-5D-5L and EQ-VAS scales. Functional performance was additionally assessed using the 30 s chair stand test and the Modified Borg Scale. Multivariable linear regression and logistic regression analyses were performed to identify predictors of reduced quality of life and persistent pain at 8 months. **Results:** A total of 80 patients were included in the study. Median VAS score decreased from 3.0 (0–6) at 1 month to 0 (0–0) at 4 months and 1 (0–1) at 8 months (*p* < 0.001). Median LANSS score decreased from 0 (0–2) at 1 month to 0 (0–0) at 4 and 8 months (*p* < 0.001), while the proportion of patients with LANSS ≥ 12 declined from 11.3% to 2.5%. EQ-5D index scores improved from 0.81 (0.72–0.91) to 0.93 (0.88–0.96) during follow-up (*p* < 0.001). Dyspnea severity, exertional symptoms, and functional limitation also improved significantly over time. Most of the clinical recovery occurred between the 1-month and 4-month evaluations, although smaller but significant improvements continued until 8 months for pain severity, functional performance, and quality-of-life measures. Older age, baseline dyspnea, anxiety, and higher baseline LANSS scores were independently associated with lower EQ-VAS scores at 8 months, whereas only higher baseline LANSS scores remained independently associated with persistent pain. **Conclusions:** Patients hospitalized with COVID-19 experience persistent pain, dyspnea, neuropathic symptoms, and reduced quality of life during follow-up, although most symptoms improve substantially over time. Early neuropathic symptom burden and baseline dyspnea may help identify patients at risk of poorer long-term recovery, although the associations should be interpreted cautiously.

## 1. Introduction

The coronavirus disease 2019 (COVID-19) pandemic has resulted in an unprecedented global health crisis, affecting millions of individuals worldwide [1]. Although the acute manifestations of severe acute respiratory syndrome coronavirus-2 (SARS-CoV-2) infection primarily involve the respiratory system, increasing evidence has demonstrated that the disease may lead to a wide range of systemic complications affecting multiple organ systems [2,3]. As the pandemic progressed, it became evident that a considerable proportion of patients continued to experience persistent symptoms even after recovery from the acute infection [4]. This condition, commonly referred to as post-acute COVID-19 syndrome or “long COVID,” has emerged as an important clinical entity with substantial implications for long-term patient outcomes [5].

Among the various symptoms reported in post-COVID syndrome, chronic pain has received increasing attention. Patients recovering from COVID-19 frequently report musculoskeletal pain, fatigue, and neurological symptoms months after the initial infection [6,7]. Neuropathic pain has been recognized as a significant component of post-COVID symptomatology [8]. Viral infections are known to induce neuropathic pain through several mechanisms, including neuroinflammation, immune-mediated neuronal injury, and cytokine-induced sensitization of nociceptive pathways [9,10]. In the case of SARS-CoV-2 infection, the neuroinvasive potential of the virus and the intense inflammatory response generated during infection may contribute to the development of persistent neuropathic symptoms [11,12].

Another major concern following COVID-19 infection is the impairment of health-related quality of life (HRQoL). Persistent symptoms such as dyspnea, fatigue, and pain may significantly limit physical functioning and daily activities [13,14]. Previous studies have demonstrated that patients who experienced severe COVID-19, particularly those requiring hospitalization, are at increased risk of long-term functional limitations and reduced quality of life [15]. Respiratory symptoms, especially dyspnea, appear to play a particularly important role in determining long-term patient outcomes [16,17].

Persistent symptoms following acute COVID-19 infection may involve heterogeneous mechanisms, including residual systemic inflammation, immune dysregulation, small-fiber neuropathy, central sensitization, and post-intensive care syndrome [18,19]. Fernández-de-Las-Peñas et al. [20] proposed an integrative classification for post-COVID symptom persistence, distinguishing between exacerbation of pre-existing conditions, persistent symptoms directly related to acute infection, and delayed-onset manifestations emerging after recovery. Within this framework, persistent pain and neuropathic symptoms may reflect both peripheral nervous system involvement and broader biopsychosocial consequences of prolonged illness [21]. Previous longitudinal studies have shown that pain, dyspnea, fatigue, and reduced quality of life may persist for several months after hospitalization for COVID-19 [22,23]. Neuropathic symptoms have attracted increasing attention because of their potential association with persistent inflammation, small-fiber nerve injury, and altered central pain processing [21,24]. However, most previous studies have been cross-sectional, have focused on single symptom domains, or have included limited follow-up durations.

Despite the growing body of literature on long COVID, the factors that predict persistent pain and reduced quality of life after hospitalization remain incompletely understood [25]. In particular, the prognostic role of early neuropathic symptoms has not been sufficiently explored in longitudinal follow-up studies. Identifying early predictors of long-term symptom persistence may help clinicians recognize high-risk patients and guide early interventions [26,27].

Therefore, the present study aimed to investigate the longitudinal course of pain, neuropathic symptoms, dyspnea, functional limitation, and health-related quality of life in previously hospitalized COVID-19 patients and to identify factors associated with persistent pain and reduced quality of life at 8 months.

## 2. Materials and Methods

### 2.1. Study Design and Participants

This study was designed as a longitudinal cohort study evaluating the progression of pain, neuropathic symptoms, and health-related quality of life in patients previously hospitalized with COVID-19. The study was conducted at Gazi Yaşargil Training and Research Hospital, Diyarbakır, Türkiye. Ethical approval was obtained from the Clinical Research Ethics Committee of Gazi Yaşargil Training and Research Hospital (approval date: 3 September 2021, approval number: 891). Patients hospitalized with polymerase chain reaction-confirmed COVID-19 infection between September 2021 and February 2022 were screened for eligibility. Patients aged ≥18 years who completed follow-up evaluations at approximately 1, 4, and 8 months after discharge were included in the study. The first post-discharge assessment, performed approximately 1 month after hospital discharge, was accepted as the baseline follow-up evaluation for the present analysis. Patients with pre-existing neurological disorders, previously diagnosed neuropathic pain syndromes, fibromyalgia, inflammatory rheumatic diseases, severe psychiatric disorders, malignancy, chronic inflammatory diseases, advanced renal or hepatic failure, or cognitive impairment preventing completion of questionnaires were excluded. Patients with incomplete clinical data or missing follow-up assessments were also excluded.

Clinical data regarding the severity of acute COVID-19 infection were collected from hospital records at Gazi Yaşargil Training and Research Hospital only by Dr. Murat Baloğlu, including hospitalization duration, oxygen requirement, intensive care unit admission, corticosteroid treatment, and mechanical ventilation support. In addition, information regarding comorbid diseases, smoking status, body mass index, vaccination status, previous chronic pain history, analgesic use during follow-up, anxiety, and depression was recorded. Follow-up assessments were performed during scheduled outpatient visits and were supplemented by structured telephone interviews when in-person evaluation was not possible. Questionnaire-based assessments were completed either face-to-face or by telephone, whereas functional tests were performed only during outpatient visits. During follow-up, patients completed standardized questionnaires regarding pain, neuropathic symptoms, dyspnea, functional capacity, and quality of life. Written informed consent was obtained from all participants before enrollment in the follow-up assessments. All patient data were anonymized before analysis, and confidentiality was maintained in accordance with institutional and national ethical standards.

**Figure 1:**
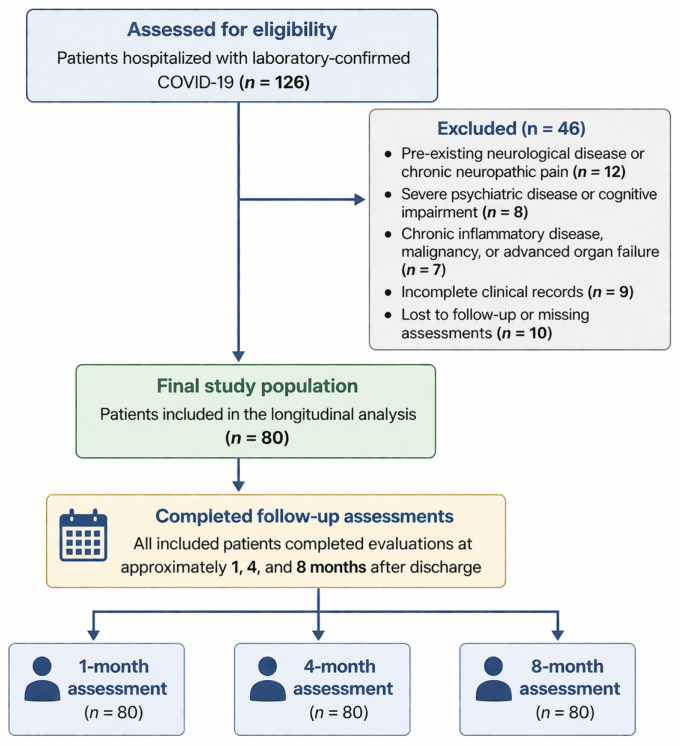
Flow diagram of patient selection and follow-up process.

A total of 126 patients hospitalized with laboratory-confirmed COVID-19 were screened for eligibility (Figure 1). Among these, 46 patients were excluded: 12 had pre-existing neurological disease or chronic neuropathic pain; 8 had severe psychiatric disease or cognitive impairment; 7 had chronic inflammatory disease, malignancy, or advanced organ failure; 9 had incomplete clinical records; and 10 were lost to follow-up or had missing assessments. Consequently, 80 patients were included in the final longitudinal analysis. All included patients completed evaluations at approximately 1, 4, and 8 months after discharge.

### 2.2. Clinical Assessment and Outcome Measures

Multiple validated clinical instruments were used to assess pain severity, neuropathic symptoms, dyspnea, functional limitation, exertional symptoms, and health-related quality of life during follow-up evaluations.

### 2.3. Pain and Neuropathic Pain Assessment

Pain intensity was assessed using the Visual Analog Scale (VAS) [28]. The VAS is a widely used instrument that measures pain intensity on a scale ranging from 0 (no pain) to 10 (worst imaginable pain). VAS scores were recorded at the 1-, 4-, and 8-month follow-up visits. Persistent pain was primarily defined as a VAS score > 0 at the 8-month evaluation. In addition, a sensitivity analysis was performed using a more clinically meaningful threshold of VAS ≥ 3. Neuropathic pain symptoms were evaluated using the Leeds Assessment of Neuropathic Symptoms and Signs (LANSS) scale [29]. The LANSS questionnaire is a validated screening tool designed to identify neuropathic pain based on symptom characteristics and sensory findings. Scores range from 0 to 24, with values ≥12 indicating a likely neuropathic pain component. LANSS scores were recorded at each follow-up visit, and the proportion of patients with LANSS ≥ 12 was additionally calculated.

### 2.4. Health-Related Quality of Life

Health-related quality of life was assessed using the EuroQol 5-Dimension 5-Level questionnaire (EQ-5D-5L) [30] and the EQ-VAS scale [31]. The EQ-5D-5L evaluates five dimensions of health status, including mobility, self-care, usual activities, pain/discomfort, and anxiety/depression. Responses were converted into a summary index score, with higher scores indicating better health status. The EQ-VAS scale measures the patient’s overall perceived health status on a scale ranging from 0 to 100, with higher values indicating better perceived health-related quality of life. EQ-VAS score at 8 months was used as the dependent variable in the multivariable linear regression analysis.

### 2.5. Dyspnea and Functional Status Assessment

Respiratory symptoms were evaluated using the modified Medical Research Council (mMRC) dyspnea scale [32], which categorizes dyspnea severity according to the degree of activity limitation. Scores range from 0 to 4, with higher scores indicating more severe dyspnea. Functional status was assessed using the Post-COVID Functional Status (PCFS) scale [33], the 30 s chair stand test (30CST) [34], and the Modified Borg Scale (MBS) [35]. The PCFS scale was used to evaluate the impact of post-COVID symptoms on daily activities and functional limitations, with scores ranging from 0 to 4 and higher scores indicating greater impairment. The 30 s chair stand test was used to evaluate lower extremity functional performance and endurance. Participants were instructed to stand up and sit down from a chair without using their arms for support as many times as possible within 30 s, and the total number of completed repetitions was recorded. The Modified Borg Scale was used to assess perceived exertion during physical activity. Scores range from 0 to 10, with higher scores indicating greater subjective exertion and respiratory discomfort.

### 2.6. Follow-Up Procedure

Participants were evaluated at approximately 1, 4, and 8 months after hospitalization. At each follow-up visit, pain severity, neuropathic symptoms, dyspnea severity, exertional symptoms, functional limitation, and quality-of-life measures were recorded using standardized questionnaires and functional assessments. Follow-up assessments were primarily conducted during outpatient visits and supplemented by structured telephone interviews when in-person evaluation was not feasible.

### 2.7. Statistical Analysis

Statistical analyses were performed using IBM SPSS Statistics version 25 (IBM Corp., Armonk, NY, USA). Continuous variables were presented as mean ± standard deviation or median with interquartile range (IQR) according to data distribution, whereas categorical variables were expressed as frequencies and percentages. Normality was assessed using the Shapiro–Wilk test. Longitudinal changes in pain severity, neuropathic symptoms, dyspnea, functional status, and quality-of-life measures across the 1-, 4-, and 8-month follow-up visits were evaluated using the Friedman test for repeated non-parametric measurements. Effect size for longitudinal analyses was estimated using Kendall’s W coefficient. When the Friedman test demonstrated statistical significance, post hoc pairwise comparisons between time points were performed using the Wilcoxon signed-rank test with Bonferroni correction. These comparisons included the 1-month versus 4-month, 1-month versus 8-month, and 4-month versus 8-month assessments.

To identify factors associated with reduced quality of life at 8 months, multivariable linear regression analysis was performed using EQ-VAS score at 8 months as the dependent variable. Variables entered into the model included age, sex, smoking status, comorbidity, baseline dyspnea, baseline VAS score, baseline LANSS score, anxiety, and depression. Prior to model construction, the distribution of EQ-VAS scores, residual plots, multicollinearity, and homoscedasticity assumptions were evaluated. Unstandardized regression coefficients (B), standardized beta coefficients (β), standard errors, and *p* values were reported. Model performance was evaluated using the F statistic and adjusted R^2^ value.

Multivariable logistic regression analysis was performed to identify factors associated with persistent pain at 8 months. Persistent pain at 8 months was primarily defined as a VAS score > 0 at the 8-month follow-up assessment. Odds ratios (ORs), 95% confidence intervals (CIs), and *p* values were reported. Model calibration and discrimination were assessed using the Hosmer–Lemeshow goodness-of-fit test, Nagelkerke R^2^, and area under the receiver operating characteristic curve (AUC). Given the limited sample size and the risk of model overfitting, the number of variables included in the logistic regression model was restricted according to the number of events observed at the final follow-up assessment. The events-per-variable ratio was evaluated before model construction. In addition, a sensitivity analysis was performed for clinically meaningful persistent pain, defined as a VAS score ≥ 3 at 8 months. Due to the limited number of events, only selected baseline variables, including baseline VAS score, baseline LANSS score, baseline dyspnea, and anxiety, were included in this exploratory analysis.

Because repeated measurements were obtained from the same participants over time, Friedman tests with Bonferroni-corrected post hoc comparisons were preferred for longitudinal analyses. Mixed-effects modeling was not performed because the primary aim of the study was to describe overall symptom trajectories and identify factors associated with outcomes at the final follow-up assessment. A *p* value < 0.05 was considered statistically significant.

## 3. Results

### 3.1. Characteristics of the Study Cohort

The baseline demographic and clinical characteristics of the study population are summarized in Table 1. A total of 80 patients were included in the study. The mean age of the cohort was 40.7 ± 13.0 years, and 52.5% of the participants were female. Comorbid diseases were present in 38.8% of patients, while 27.5% reported baseline dyspnea. During the acute hospitalization period, 11.3% of patients required ICU admission, 35.0% received oxygen support, and 3.8% underwent mechanical ventilation. Corticosteroid treatment was administered to 40.0% of patients, and the median duration of hospitalization was 8 days. A history of chronic pain was reported by 15.0% of patients, whereas 36.3% used analgesics during follow-up. Baseline median VAS and LANSS scores were 3.0 (0–6) and 0 (0–2), respectively.

### 3.2. Longitudinal Changes in Pain, Neuropathic Symptoms, Functional Capacity, and Quality of Life

Longitudinal changes in pain severity, neuropathic symptoms, dyspnea, functional status, and quality-of-life measures across the three follow-up assessments are presented in Table 2. For longitudinal outcomes, pain severity, neuropathic symptoms, dyspnea, exertional symptoms, and functional limitations improved significantly over time. Median VAS score decreased from 3.0 (0–6) at 1 month to 0 (0–0) at 4 months, but increased slightly to 0 (0–1) at 8 months, indicating an irregular symptom trajectory rather than a continuously improving pattern. Similarly, median LANSS score declined from 0 (0–2) at 1 month to 0 (0–0) at both 4 and 8 months. The proportion of patients with clinically relevant neuropathic pain features also decreased over time. EQ-5D index scores increased progressively throughout follow-up, whereas mMRC dyspnea scores, Modified Borg Scale scores, and PCFS scores decreased significantly. Functional performance, as measured by the 30 s chair stand test, improved steadily over time. Although EQ-VAS scores remained generally high throughout follow-up, a statistically significant overall difference was observed across time points.

### 3.3. Temporal Patterns of Recovery Across Follow-Up Assessments

Post hoc pairwise comparisons between follow-up time points are summarized in Table 3. Post hoc pairwise comparisons demonstrated that most of the clinical improvement occurred during the early recovery period between the 1-month and 4-month follow-up assessments. Significant reductions in VAS score, LANSS score, mMRC dyspnea score, PCFS score, and Modified Borg Scale score were observed between 1 and 4 months, and these improvements remained significant at the 8-month assessment. Similarly, EQ-VAS and EQ-5D index scores increased significantly over time, while performance on the 30 s chair stand test improved progressively.

Between the 4-month and 8-month evaluations, some parameters continued to show significant improvement, although the magnitude of change was smaller. VAS score, Modified Borg Scale score, EQ-5D index score, and 30 s chair stand test performance remained significantly improved between these time points. In contrast, no significant differences were observed between 4 and 8 months for LANSS score, EQ-VAS score, mMRC dyspnea score, or PCFS score, suggesting stabilization of neuropathic symptoms, dyspnea, and functional status after the intermediate recovery period.

### 3.4. Predictors of Reduced Quality of Life at 8 Months

The results of the multivariable linear regression analysis for EQ-VAS score at 8 months are presented in Table 4. Lower EQ-VAS scores at 8 months were independently associated with older age, baseline dyspnea, higher baseline LANSS scores, and anxiety. In the multivariable model, age (β = −0.31, *p* = 0.001), baseline dyspnea (β = −0.30, *p* = 0.002), baseline LANSS score (β = −0.31, *p* = 0.004), and anxiety (β = −0.32, *p* = 0.001) remained significant independent predictors of poorer self-perceived health status at long-term follow-up. The overall model explained 42.0% of the variance in EQ-VAS scores at 8 months (adjusted R^2^ = 0.420).

Regression diagnostics were examined before the multivariable linear regression analysis. No substantial multicollinearity was detected among the predictors, with all variance inflation factor values below 1.5. The Durbin–Watson statistic was 1.64, indicating no major autocorrelation of residuals. However, EQ-VAS scores showed a marked ceiling distribution, with median values of 100 at all follow-up assessments, and residual normality was imperfect. Therefore, although the regression assumptions were checked and considered acceptable overall, the clinical and statistical interpretation of the linear regression model should be approached cautiously.

### 3.5. Predictors of Persistent Pain at 8 Months

The multivariable logistic regression analysis identifying predictors of persistent pain at 8 months is summarized in Table 5. Persistent pain at 8 months was observed in 48 patients (60.0%). In the multivariable model, higher baseline LANSS score was the only variable that remained significantly associated with persistent pain at 8 months. However, this finding should be interpreted cautiously given the limited number of persistent pain events and the low prevalence of elevated LANSS scores in the cohort (OR = 5.75, 95% CI 1.88–17.60, *p* = 0.002). None of the other evaluated variables, including age, sex, smoking status, comorbidity, baseline dyspnea, anxiety, or depression, remained statistically significant in the adjusted model. The regression model demonstrated acceptable calibration and discrimination (Hosmer–Lemeshow *p* = 0.68, Nagelkerke R^2^ = 0.301, AUC = 0.872).

### 3.6. Exploratory Analysis of Clinically Meaningful Persistent Pain

The exploratory analysis evaluating predictors of clinically meaningful persistent pain is presented in Table 6. Only three patients met the clinically meaningful persistent pain threshold, defined as VAS ≥ 3 at 8 months. Exploratory univariable analysis showed that higher baseline LANSS scores were associated with an increased likelihood of clinically meaningful persistent pain (OR = 2.92, 95% CI 1.08–7.91, *p* = 0.034). However, only three patients met the VAS ≥ 3 threshold at 8 months. Therefore, these findings should be interpreted cautiously due to the very limited number of events, lack of statistical power, and exploratory nature of the analysis. Table 6 presents the exploratory analysis results.

## 4. Discussion

The transition from acute SARS-CoV-2 infection to post-COVID syndrome represents a complex clinical phenomenon characterized by persistent neurological symptoms, chronic pain, dyspnea, functional limitation, and impaired health-related quality of life (HRQoL) [36,37,38]. Although most patients demonstrate gradual clinical recovery following the acute phase of infection, accumulating evidence suggests that a substantial subset of individuals experience long-term sequelae that may persist for months after hospitalization [39,40]. These persistent manifestations highlight the multifactorial pathophysiology of post-COVID syndrome and its potential impact on long-term functional outcomes. In the present study, pain severity, neuropathic symptoms, dyspnea, exertional symptoms, functional limitation, and HRQoL improved progressively over the 8-month follow-up period. The most pronounced improvements were observed between the first- and fourth-month evaluations, whereas smaller but still significant changes continued until the eighth month. However, recovery was not completely linear. Pain severity decreased markedly during the early follow-up period but showed a slight increase at the 8-month evaluation. This finding suggests that some patients may experience fluctuating or persistent symptoms despite initial improvement, a pattern that has also been reported in previous long COVID cohorts.

One of the most important aspects of post-COVID symptomatology is the development of neuropathic pain [41]. The biological mechanisms underlying persistent neuropathic symptoms after viral infections are complex and involve direct viral neurotropism, immune dysregulation, neuroinflammatory responses, and small-fiber nerve injury [42]. Zubair et al. [43] reported that SARS-CoV-2 has demonstrated neuroinvasive potential, partly mediated through the expression of angiotensin-converting enzyme 2 (ACE2) receptors within neuronal and glial cells of the nervous system. Mehta et al. [44] showed that viral invasion and systemic infection trigger the release of pro-inflammatory cytokines, including tumor necrosis factor-α (TNF-α), interleukin-1β (IL-1β), and interleukin-6 (IL-6), which are key mediators of systemic immune activation. Furthermore, this cytokine-mediated inflammatory cascade has been shown to sensitize both peripheral and central nociceptive pathways, potentially contributing to the development of persistent neuropathic pain after COVID-19 infection [45]. Additionally, endothelial injury and microthrombosis affecting the vasa nervorum may contribute to ischemic neuropathy and small-fiber nerve damage observed in post-COVID patients [46].

Recent studies have increasingly reported a neuropathic component in post-COVID pain syndromes. Herrero-Montes et al. [47,48] reported that approximately one-quarter of previously hospitalized COVID-19 survivors experiencing persistent pain exhibit neuropathic characteristics based on screening tools such as the S-LANSS questionnaire. In addition, prolonged hospitalization and intensive care management may further contribute to chronic pain through mechanisms associated with post-intensive care syndrome, including immobilization, critical illness neuropathy, and muscle weakness [49]. These findings suggest that persistent pain after COVID-19 may result from the combined effects of viral neuroinvasion, systemic inflammation, and hospitalization-related complications [50].

The temporal trajectory of neuropathic symptoms observed in our study is consistent with the hypothesis that early inflammatory neural injury may partially resolve over time [51]. LANSS scores declined substantially during the first months of follow-up and appeared to stabilize thereafter. However, despite the low median LANSS values observed throughout follow-up, patients with higher baseline LANSS scores were more likely to report persistent pain at 8 months. This finding suggests that clinically relevant neuropathic symptoms were concentrated within a relatively small subgroup of patients rather than being uniformly distributed across the entire cohort. Therefore, the present findings should not be interpreted as evidence that neuropathic mechanisms are universally present in all post-COVID patients, but rather that patients with greater early neuropathic symptom burden may be at increased risk for persistent pain [52].

In addition to pain-related symptoms, functional recovery following COVID-19 infection appears to be strongly influenced by demographic factors, dyspnea severity, and psychological status. In the present study, older age, baseline dyspnea, higher baseline LANSS score, and anxiety were independently associated with poorer long-term HRQoL. In contrast, only baseline LANSS score remained independently associated with persistent pain at 8 months. These findings are consistent with previous studies showing that respiratory symptoms, anxiety, and neuropathic pain symptoms often coexist during post-COVID recovery and may interact through complex biopsychosocial mechanisms [53,54,55].

The functional findings of the present study further support the multidimensional impact of post-COVID syndrome. Significant improvements were observed in mMRC dyspnea scores, PCFS scores, Modified Borg Scale scores, and 30 s chair stand test performance throughout follow-up. These findings suggest that recovery after COVID-19 involves not only reduction in pain burden but also gradual improvement in respiratory symptoms, exercise tolerance, and functional independence. Nevertheless, a proportion of patients continued to report dyspnea, exertional limitation, and impaired daily functioning at 8 months, emphasizing that full recovery may be delayed in some individuals [56]. Health-related quality of life improved significantly throughout follow-up according to both EQ-5D-5L and EQ-VAS assessments [57,58]. However, EQ-VAS values remained relatively high throughout the study period. This distribution suggests a possible ceiling effect, meaning that the EQ-VAS instrument may have had limited sensitivity to detect small but clinically meaningful changes in patients with relatively preserved health status. Therefore, the statistically significant improvements observed in EQ-VAS scores should be interpreted cautiously.

The present findings should also be interpreted in the context of possible residual confounding. Acute COVID-19 severity, intensive care unit admission, mechanical ventilation, corticosteroid use, hospitalization duration, pre-existing chronic pain, and vaccination status may all influence long-term pain trajectories and quality of life. Although several clinically relevant variables were included in the multivariable analyses, unmeasured confounders may still have affected the observed associations. Therefore, causal inferences cannot be made from the present findings.

The identification of early neuropathic symptoms may still be clinically relevant in post-COVID follow-up care. Routine screening using validated instruments such as the LANSS or S-LANSS questionnaires may help identify patients who are more likely to experience persistent pain during recovery [59]. However, unlike interventional studies, the present observational study cannot support specific pharmacological recommendations. Although medications such as gabapentinoids or serotonin–norepinephrine reuptake inhibitors may theoretically be considered in selected patients with neuropathic symptoms, future prospective and interventional studies are required before such approaches can be recommended routinely.

The present study has several limitations. First, it was conducted in a single center with a relatively small sample size, which may limit the generalizability of the findings. Second, although multiple variables were included in the regression analyses, the limited number of persistent pain events may have increased the risk of model overfitting. In addition, neuropathic pain symptoms were relatively uncommon in this cohort, with median LANSS scores of 0 at all time points and only a small proportion of participants reaching the conventional LANSS ≥ 12 threshold. Therefore, the observed association between baseline LANSS score and persistent pain may be statistically unstable and should be interpreted cautiously. Third, detailed neurological examination findings, inflammatory biomarkers, imaging data, and neurophysiological assessments were not available. Therefore, the biological mechanisms underlying persistent pain and neuropathic symptoms could not be directly evaluated. Fourth, pain location and pain phenotype were not systematically categorized; therefore, it could not be determined whether persistent symptoms were predominantly musculoskeletal, neuropathic, or mixed in origin. Fifth, EQ-VAS scores demonstrated a possible ceiling effect, which may have reduced the sensitivity of quality-of-life analyses. Finally, although repeated measurements were available, mixed-effects models were not used, and residual confounding due to unmeasured variables cannot be excluded.

## 5. Conclusions

In conclusion, pain severity, neuropathic symptoms, dyspnea, functional limitation, and health-related quality of life generally improved during recovery after COVID-19 hospitalization. However, a subgroup of patients continued to experience persistent symptoms at long-term follow-up. Higher early neuropathic symptom burden was associated with persistent pain at 8 months, whereas older age and baseline dyspnea were associated with poorer long-term quality of life. These findings highlight the importance of early recognition of neuropathic symptoms, respiratory impairment, and psychological factors during post-COVID follow-up. Nevertheless, because of the observational nature of the study, causal inferences cannot be made. Future prospective studies with larger cohorts and more detailed neurological and functional assessments are needed to better define the mechanisms underlying persistent post-COVID symptoms and to identify patients at greatest risk for long-term morbidity.

## Figures and Tables

**Table 1:** Baseline Clinical Characteristics of the Study Population.

Variable	Value
Number of patients	80
Age, mean ± SD	40.7 ± 13.0
Age, median (IQR)	39.0 (28.5–49.5)
Male sex, *n* (%)	38 (47.5%)
Female sex, *n* (%)	42 (52.5%)
Comorbidity present, *n* (%)	31 (38.8%)
Baseline dyspnea, *n* (%)	22 (27.5%)
ICU admission during hospitalization, *n* (%)	9 (11.3%)
Oxygen support, *n* (%)	28 (35.0%)
Mechanical ventilation, *n* (%)	3 (3.8%)
Corticosteroid treatment, *n* (%)	32 (40.0%)
Length of hospitalization, days, median (IQR)	8 (5–12)
Vaccinated before infection, *n* (%)	21 (26.3%)
History of chronic pain, *n* (%)	12 (15.0%)
Analgesic use during follow-up, n (%)	29 (36.3%)
Smoking status, *n* (%)	14 (17.5%)
Body mass index, mean ± SD	25.6 ± 4.1
Alcohol use, *n* (%)	1 (1.3%)
Depression, *n* (%)	14 (17.5%)
Anxiety, *n* (%)	15 (18.8%)
EQ-VAS score at 1 month	100 (100–100)
Baseline LANSS score	0 (0–2)
Baseline VAS score	3.0 (0–6)

Values are presented as mean ± standard deviation, median (interquartile range), or number (percentage), as appropriate. Baseline evaluation corresponds to the first follow-up assessment performed approximately 1 month after hospital discharge. Comorbidity refers to the presence of at least one chronic medical condition. Anxiety and depression were based on previously documented clinical diagnosis or current treatment.

**Table 2:** Longitudinal changes in pain, neuropathic symptoms, functional status, and quality of life.

Variable	1 Month	4 Months	8 Months	*p* Value	Kendall’s W
VAS score	3.0 (0–6)	0 (0–0)	1 (0–1)	<0.001	0.282
LANSS score	0 (0–2)	0 (0–0)	0 (0–0)	<0.001	0.444
Patients with LANSS ≥ 12, n (%)	9 (11.3%)	3 (3.8%)	2 (2.5%)	0.002	0.228
EQ-VAS score	100 (100–100)	100 (90–100)	100 (90–100)	<0.001	0.115
EQ-5D index score	0.81 (0.72–0.91)	0.91 (0.84–0.95)	0.93 (0.88–0.96)	<0.001	0.238
mMRC dyspnea score	1 (0–2)	0 (0–1)	0 (0–1)	0.003	0.146
30 s chair stand test	10 (8–12)	12 (10–14)	13 (11–15)	<0.001	0.254
Modified Borg Scale	3 (2–5)	2 (1–3)	1 (0–2)	<0.001	0.301
PCFS score	2 (1–3)	1 (0–2)	1 (0–2)	<0.001	0.201

Values are presented as median (interquartile range) or number (percentage), as appropriate. Longitudinal comparisons were performed using the Friedman test for repeated measurements. Effect size was estimated using Kendall’s W coefficient. Post hoc pairwise comparisons were performed using the Wilcoxon signed-rank test with Bonferroni correction. LANSS scores ≥ 12 were considered indicative of neuropathic pain.

**Table 3:** Post Hoc Pairwise Comparisons of Longitudinal Outcomes.

Variable	1 Month vs. 4 Months	1 Month vs. 8 Months	4 Months vs. 8 Months
VAS score	<0.001	<0.001	<0.001
LANSS score	<0.001	<0.001	0.157
EQ-VAS score	0.001	*p* < 0.001	0.276
mMRC dyspnea score	0.004	0.011	0.421
PCFS score	<0.001	<0.001	0.089
Modified Borg Scale	<0.001	<0.001	0.018
EQ-5D index score	<0.001	<0.001	0.041
30 s chair stand test	<0.001	<0.001	0.006

Post hoc pairwise comparisons were performed using the Wilcoxon signed-rank test with Bonferroni correction. *p* values < 0.05 were considered statistically significant after adjustment for multiple comparisons.

**Table 4:** Multivariable Linear Regression Analysis for EQ-VAS Score at 8 Months.

Variable	B	Standardized β	Standard Error	*p* Value
Age	−0.37	−0.31	0.11	0.001
Female sex	−1.12	−0.04	2.89	0.700
Smoking	−0.82	−0.02	3.74	0.827
Comorbidity	0.30	0.01	3.35	0.928
Baseline dyspnea	−10.34	−0.30	3.16	0.002
Baseline VAS score	−0.73	−0.14	0.51	0.153
Baseline LANSS score	−2.14	−0.31	0.72	0.004
Anxiety	−12.67	−0.32	3.71	0.001
Depression	0.59	0.01	3.57	0.869

Multivariable linear regression analysis was performed using EQ-VAS score at 8 months as the dependent variable. Variables entered into the model included age, sex, smoking status, comorbidity, baseline dyspnea, baseline VAS score, baseline LANSS score, anxiety, and depression. B: unstandardized regression coefficient; β: standardized regression coefficient; SE: standard error; CI: confidence interval.

**Table 5:** Multivariable Logistic Regression Analysis for Persistent Pain at 8 Months (VAS > 0).

Variable	OR	95% CI	*p* Value
Age	1.01	0.97–1.05	0.723
Female sex	1.10	0.34–3.58	0.875
Smoking	1.54	0.39–6.04	0.538
Comorbidity	2.60	0.57–11.82	0.215
Baseline dyspnea	1.53	0.40–5.86	0.533
Baseline VAS score	0.99	0.79–1.22	0.893
Baseline LANSS score	5.75	1.88–17.60	0.002
Anxiety	1.02	0.24–4.42	0.977
Depression	0.67	0.14–3.17	0.609

Persistent pain was defined as a VAS score > 0 at the 8-month follow-up assessment. Odds ratios (ORs), 95% confidence intervals (CIs), and *p* values are presented.

**Table 6:** Sensitivity Analysis for Clinically Meaningful Persistent Pain (VAS ≥ 3).

Variable	OR	95% CI	*p* Value
Baseline VAS score	1.48	0.92–2.39	0.102
Baseline LANSS score	2.92	1.08–7.91	0.034
Baseline dyspnea	2.17	0.39–12.18	0.376
Anxiety	2.44	0.41–14.63	0.326

Clinically meaningful persistent pain was defined as a VAS score ≥ 3 at the 8-month follow-up assessment. Due to the limited number of events, only selected baseline variables were included in the exploratory model. Odds ratios (ORs), 95% confidence intervals (CIs), and *p* values are presented.

## Data Availability

The data presented in this study are available on request from the corresponding author. The data are not publicly available due to privacy and ethical restrictions.
